# On the Path to Measles and Rubella Elimination Following Rubella-Containing Vaccine Introduction, 2000–2023, Namibia

**DOI:** 10.3390/vaccines12090957

**Published:** 2024-08-23

**Authors:** Balcha G. Masresha, Messeret E. Shibeshi, Roselina de Wee, Nicholas Shapumba, Takudzwa Sayi, Susan E. Reef, James L. Goodson

**Affiliations:** 1 African Regional Office, World Health Organization, Brazzaville P.O. Box 06, Congo; eshetum@who.int; 2 Country Office, World Health Organization, Windhoek P.O. Box 3444, Namibia; deweer@who.int; 3 Expanded Programme on Immunization, Namibia Ministry of Health and Social Services, Windhoek 13198, Namibia; 4 Global Immunization Division, US Centers for Disease Control and Prevention, Atlanta, GA 30333, USA; prp2@cdc.gov (T.S.); fez9@cdc.gov (J.L.G.); 5 Independent Consultant, Atlanta, GA 30329, USA

**Keywords:** measles, rubella, Namibia, incidence, elimination

## Abstract

Introduction: The WHO Measles and Rubella Strategic Framework 2021–2030 within the Immunization Agenda 2030 includes both measles and rubella elimination goals and provides guidance to countries for planning and implementing the measles and rubella elimination strategies. Namibia has been implementing measles elimination strategies since 1997. Methods: We reviewed and described the implementation of measles and rubella elimination strategies and the programmatic and epidemiological situation in Namibia during 2000–2023. Namibia introduced a rubella-containing vaccine (RCV) in 2016 as a combined measles–rubella (MR) vaccine using a MR catch-up campaign, targeting a wide age range based on detailed analysis and triangulation of multiple key data sources including MR vaccination coverage, MR case-based surveillance, detailed measles outbreak investigations, and serosurveys. Results: In 2020, estimated MCV1 coverage in Namibia reached 90% and has been sustained at 91% in 2021 and 2022. MCV2 was introduced in 2016, and the estimated MCV2 coverage has steadily increased to 79% in 2022. Following the MCV2 introduction and the implementation of the wide age range MR catch-up campaign in 2016, annual measles and rubella incidence decreased substantially. During 2017–2023, the period following the implementation of the catch-up MR vaccination SIA in 2016, average annual measles incidence per million population in Namibia decreased by 97% from the average during 2010–2016. Similarly, the average annual rubella incidence decreased by 95% from 2010–2016 to 2017–2023. Discussion: Successful implementation of the 2016 wide age range campaign and maintaining high routine immunization coverage likely led to the significant reduction in measles and rubella incidence in Namibia. To sustain the reduction in measles and rubella incidence and attain the elimination targets, Namibia needs to attain and maintain high routine immunization coverage with both doses of the MR vaccine and implement timely and high-quality periodic MR follow-up SIAs. High-quality elimination-standard measles and rubella surveillance will help guide strategies and serve as the basis for the eventual verification of measles and rubella elimination in Namibia according to the WHO-recommended framework.

## 1. Introduction

Measles remains a major cause of child mortality worldwide, and rubella is the leading cause of birth defects among all infectious diseases globally [1,2,3,4], despite the availability of safe and effective measles and rubella vaccines since the 1960s. Both diseases are among a handful that have been identified for possible eradication [5,6]. The feasibility and benefits of measles and rubella elimination are well established, and global progress toward elimination continues to be made [7,8,9,10,11,12]. Elimination of both diseases is optimally achieved by implementing synergistic integrated strategies that include using an inexpensive, safe, and highly effective combined measles and rubella vaccine, case-based disease surveillance for rash-fever illness to detect and confirm both diseases, and rapid outbreak response [13]. To introduce a rubella-containing vaccine (RCV) in a national immunization program, the WHO recommends first using a combined measles–rubella (MR) vaccine in a national wide age range catch-up campaign and then using the MR vaccine for all recommended vaccination opportunities, including the two routine doses, periodic SIAs, and measles outbreak response immunizations (ORIs). During measles ORIs, using MR is critically important since measles is a tracer for identifying areas of weakness in immunization services, low vaccination coverage, and immunity gaps for both measles and rubella [14,15,16].

The basic reproductive number (R_0_) ranges from 9 to 18 for measles and from 4 to 7 for rubella, depending on context-specific variables [17,18]; recent analyses suggest that rubella R_0_ can be even lower in some settings [19]. Herd immunity thresholds for measles are higher than for rubella, and while both vaccines are highly effective, vaccine effectiveness for the rubella vaccine is slightly higher than the measles vaccine; therefore, the integrated MR elimination strategies are generally driven by measles epidemiology. Historically, national immunization programs have sequentially introduced the two vaccines, with the measles vaccine first and the rubella vaccine introduction coming some years later [18,20]. By the end of 2023, only 19 countries worldwide remained without rubella vaccine introduction, nearly all in the African Region. Rubella vaccine introduction with MR offers an important opportunity to close any remaining measles population immunity gaps while maximizing population immunity for rubella.

Global efforts to eliminate measles and rubella are ongoing. In 2012, the World Health Assembly endorsed the Global Vaccine Action Plan (GVAP), which aimed at extending the full benefits of the EPI to all and setting a target to eliminate measles and rubella in five of six WHO regions by 2020 [5,21,22,23,24]. As of 2024, all six WHO regions had established a measles elimination goal, and five a rubella elimination goal. The WHO African Region Member States established measles and rubella elimination goals in 2011 and 2021 [25,26], respectively; and the WHO provided a regional elimination strategic framework to support countries in the region [27,28].

Namibia is a WHO African Region Member State and middle-income country situated in the southwestern part of the African continent. The country shares land borders with South Africa, Botswana, Angola, and Zambia and had an estimated population of 2.5 million in 2021 [29,30]. The country is dry and sparsely inhabited with the Kalahari Desert in the east and the Namib Desert in the coastal regions; 67% of inhabitants are rural dwellers. Following the declaration of national independence in 1990, Namibia established the national Ministry of Health and Social Services (MoHSS) Expanded Programme on Immunization (EPI). Measles vaccination in the population initially began prior to independence in 1983 with the introduction of one dose of the measles vaccine given to infants at nine months of age. Starting in 2016, a routine second dose of the measles-containing vaccine (MCV2) is provided at 15 months of age through ongoing immunization services. The RCV was also introduced in 2016, following which the monovalent measles vaccine was replaced by a combined MR vaccine for all vaccination activities based on WHO recommendations [31,32,33].

During 1997–2009, measles SIAs started with a catch-up campaign in 1997 for children 9 months–14 years of age, and then periodic follow-up SIAs in 2000, 2003, 2006, and 2009 for children 9–59 months of age. Reported administrative coverage for those campaigns ranged from 89% to 104% (a value > 100% indicates that the number of doses administered exceeded the estimated target population); no post-campaign coverage surveys were implemented. Following the initial catch-up measles SIA in 1997, which reached 677,538 children aged 9 months–14 years, Namibia experienced a period of very low measles incidence for approximately five years [34,35]. After this period of very low incidence, periodic measles outbreaks with lower incidence than in the pre-vaccination era restarted, similar to patterns documented in other settings [35]. Measles outbreaks in Namibia during 2002–2003, 2009–2011, and 2013–2014 were well documented [36]. These outbreaks were characterized by shifting measles epidemiology over time that included increases in the age of infection of cases. In 2010, Ogbuanu et al. conducted a comprehensive detailed outbreak investigation and found that 38% of confirmed measles cases were in persons ≥ 15 years of age during 2009–2011 [37,38]. Additionally, an analysis of the case-based surveillance data from 2014 found that 21% of measles cases were in persons ≥ 25 years of age [39].

Seroprevalence surveys (serosurveys) were completed to further characterize population immunity for measles, rubella, and polio and to identify age groups with susceptibility for evidence to help guide measles and rubella elimination and polio eradication efforts in Namibia, [40,41,42]. These serosurveys of a randomly selectedage-stratified patient subset of previously collected and stored serum specimens from the MoHSS national HIV surveys among pregnant women aged 15–44 years and were completed in 2008 and 2010; (the results were previously published elsewhere [40,41,42]). In both serosurveys, the lowest measles seroprevalence was among persons 15–19 years of age, and overall measles seropositivity did not differ significantly between 2008 (87%) and 2010 (87%) despite a large measles outbreak occurring in the interim [40,41]. In 2010, measles seropositivity among those aged 15–19, 20–24, 25–29, 30–34, 34–39, and 40–44 years was 56%, 71%, 77%, 80%, 88%, and 91% respectively. In 2010, rubella seropositivity by 5-year age group among those aged 15–19, 20–24, 25–29, 30–34, 34–39, and 40–44 years was 83%, 86%, 82%, 86%, 88%, and 90% respectively. Women from urban residences had significantly higher rubella seroprevalence (87%) compared with women from rural residences (83%) (*p* < 0.001) [40,41].

The findings from surveillance data of increased age-specific measles incidence in older age groups, combined with the identified population immunity gaps in older age groups from the measles and rubella serosurveys, provided strong evidence of MR susceptibility in older age groups and helped define the target age group for the 2016 SIA. The epidemiological shift to older age groups following increasing vaccination coverage, a shift observed in other settings [43], was well described in Namibia during measles outbreak investigations during 2009–2010. Based on the evidence, a key decision was made by the Namibia MoHSS to use an expanded target age group in the next SIA to provide MR vaccinations for persons 9 months–39 years of age. The national MR SIA was implemented during 11–22 July 2016 and had reported administrative coverage of > 100%. It was the first opportunity to provide the RCV through the EPI in the country, and, therefore, to prevent rubella infection and make progress toward achieving rubella elimination. The SIA was followed by the introduction of the RCV in the national EPI schedule per the WHO guidance [31]. We reviewed and described the implementation of measles and rubella elimination strategies and the programmatic and epidemiological situation during 2000–2023 to document and share experiences from Namibia in advancing progress toward the goal of measles and rubella elimination.

## 2. Methods

**Routine Immunization Services:** The national estimated population was 2,133,188 in 2009 and included 69,067 surviving infants, 300,260 children < 5 years of age, and 839,112 children < 15 years of age. In 2009, life expectancy was 53 years for males and 62 years for females, and the under-five mortality rate was 69 per 1000 live births. The MoHSS is organized into three levels—national, regional, and district—with district health centers serving as the primary service delivery level. Namibian citizens who possess a registration card (called a health passport) receive all health services free of charge after payment of a nominal registration fee. In addition to the MoHSS, some immunization services are delivered through fee for services in the private sector. The national EPI provides ongoing daily immunization services at health facilities and outreach sites and during periodic SIAs at fixed post sites and by mobile teams. Vaccine doses are administered to eligible persons during daily immunization services and periodic SIAs are reported from the health facilities to the provinces and onward to the national level. Sub-national and national administrative coverage data are calculated by the EPI by dividing the number of children vaccinated during vaccination activities by the number of children in the vaccination target age group the program reports it intends to vaccinate based on estimated population denominators. The country reports the national-level coverage annually to the WHO and UNICEF as part of the annual Joint Reporting Form (JRF) process. The WHO and UNICEF use the reported administrative coverage data and any available survey results to generate the WHO/UNICEF estimates of national immunization coverage (WUENIC) for each antigen provided through routine immunization services [44]. Data from private sector immunization services were not available. We analyzed reported vaccination data for routine immunization services from the JRF and WUENIC during 2000–2023.

**Supplemental Immunization Activities:** The periodic measles and MR SIAs are implemented with the objective of achieving ≥ 95% vaccination coverage; generally, the SIAs occur every 2–5 years, depending on the rate of accumulation of measles-susceptibility, primarily based on children missed by routine immunization services [45,46]. The SIAs are designed to reach all children in the target age group and include special strategies to reach areas with large numbers of unvaccinated or “zero-dose” children [47]. The number of doses provided during SIAs is tallied at the service delivery level and compiled for reporting up to the provincial level and then the national level. The SIA administrative coverage is calculated by the EPI using the administrative method of dividing the number of children vaccinated during the SIA by the number of children in the SIA target age group for the vaccination; the denominators are based on the 2001 and 2011 census projections. SIA coverage data are reported to the WHO using official SIA technical reports. We analyzed technical reports and post-campaign surveys (PCCSs) from measles and measles–rubella SIAs implemented during 2000–2023 [48].

**Measles and Rubella Incidence and Case-based Surveillance Performance**: Nationwide measles case-based surveillance with the support of the national laboratory for serological case confirmation was established in 1998 in Namibia. Suspected measles cases were defined as illnesses characterized by a generalized maculopapular rash and fever and at least one of the following: cough, coryza, or conjunctivitis, or any case suspected to be measles by a clinician. For suspected cases, patient age, sex, address, number of measles vaccination doses received, and date of most recent measles vaccination were collected by a clinician on a standard case report form. Blood specimens were collected from persons with suspected measles cases identified within 30 days of onset and tested for measles-specific immunoglobulin M (IgM) antibodies at the national measles reference laboratory using a standard enzyme-linked immunosorbent assay. Specimens tested without a positive result were then tested for rubella-specific IgM antibodies. Confirmed measles cases were defined by either a positive result for measles IgM antibody testing, by epidemiological linkage, or clinically compatible case classification. Confirmed rubella cases were defined by a positive test result for the rubella IgM antibody. An epidemiologically linked case was defined as a case-patient with illness that met the suspected case definition and had contact (‘contact’ defined according to surveillance guidelines as living in same district or adjacent districts with plausibility of transmission) with a laboratory-confirmed measles case with rash onset within the preceding 30 days. The national case-based surveillance database and the serological laboratory database were shared by MoHSS with the WHO weekly. We analyzed the measles and rubella surveillance data from 2010 to 2023 to describe the measles and rubella epidemiology and the quality of surveillance performance measured using the WHO-recommended performance indicators. Two key performance indicators were analyzed: (1) the non-measles febrile rash illness (NMFRI) rate (target: ≥ 2 per 100,000 population) and (2) the proportion of districts that have investigated ≥ 1 suspected case of measles with blood specimen (target: ≥ 80% of districts) annually. The confirmed measles incidence per million population was calculated by dividing the total number of confirmed measles cases (confirmed by laboratory, epidemiological linkage, and clinically compatible case classification) by the total population based on the official projections from the census estimates. The incidence of confirmed rubella was calculated using the number of laboratory-confirmed rubella cases as the numerator and the same denominator as used for measles incidence [49].

### Measles Immunity Profile

Estimating measles immunity gaps can help guide immunization programs in monitoring program performance, identifying when the next SIAs should be conducted and identifying age groups that have the largest measles immunity gaps. The WHO recommends countries monitor the risk of measles outbreaks by estimating the accumulated number of measles-susceptible preschool children, and preventing outbreaks by conducting SIAs before that number reaches the equivalent of one birth cohort. This approach has been found to be programmatically useful in preventing large outbreaks. We generated the national measles immunity profile to estimate the number of persons immune by MCV1, MCV2, and SIAs and the number of measles-susceptible persons by year of birth. The estimated percentage of measles-susceptible persons is multiplied by the number of persons by age to estimate the total number of measles-susceptible persons by age as of December 31, 2023. To estimate a profile as of 31 December 2023, MCV1 and MCV2 coverage data points in 2023 were assumed to be the same as the WUENIC for 2022, the last year available. PCCS estimates were used for SIA coverage, and if unavailable, we used administrative coverage.

## 3. Results

**Figure 1:**
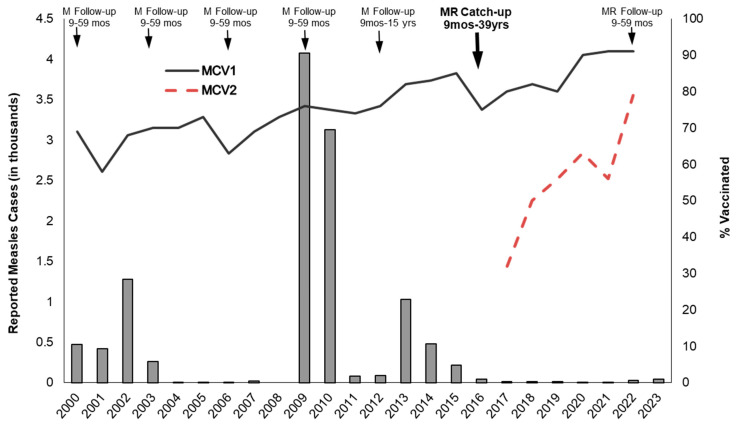
National coverage with the first dose of the measles-containing vaccine (MCV1) and second dose (MCV2), supplemental immunization activities, and reported measles cases, 2000–2023, Namibia. Notes: M = measles, MR = measles–rubella, mos = months, yrs = years; World Health Organization (WHO) and United Nations Children’s Fund (UNICEF) estimates of national immunization coverage; data from the Joint Reporting Form submitted to the WHO and UNICEF by member states with the official number of measles cases in the country for the year, http://immunizationdata.who.int (accessed on 11 June 2024).

**Ongoing Immunization Services:** Estimated MCV1 coverage in Namibia was 75% in 2010, and it steadily increased to 85% in 2015 and then fluctuated between 75% and 82% until 2019 (Figure 1). In 2020, estimated MCV1 coverage reached 90% and was sustained at 91% in both 2021 and 2022. After MCV2 was introduced in the childhood vaccination schedule in 2016, estimated MCV2 coverage steadily increased from 32% in 2017 to 79% in 2022. Since RCV introduction in 2016, the EPI has switched from using a monovalent measles vaccine to a combined MR vaccine for all doses, and RCV coverage is represented by the measles vaccination coverage data.

**Supplemental Immunization Activities:** In 2012, Namibia conducted a nationwide measles SIA for children aged 9 months–15 years (Table 1). The reported SIA administrative coverage for the 2012 SIA was 91%, and a PCCS that estimated nationwide SIA coverage was 97%. In 2016, Namibia conducted a wide age range catch-up MR SIA for persons aged 9 months–39 years as part of the strategy for RCV introduction. A total of 1,908,193 persons were vaccinated during the nationwide MR SIA, with 103% reported administrative coverage, and 77% of districts reported ≥95% administrative coverage. In 2022, a follow-up MR SIA for children aged 9–59 months was implemented with a reported 95% administrative coverage. A PCCS was not performed following SIAs in 2016 and 2022.

**Measles and Rubella Incidence:** During 2010–2016, the average annual incidence per million persons was 163.9 (range: 5.0 to 656.4) for measles and 53.3 (range: 5.0 to 172.9) for rubella. The laboratory-confirmed measles incidence per million population sharply fell from 88.3 in 2015 to 5.0 in 2016 (the year of the MR catch-up SIAs) and was 7.5 in 2017. Similarly, the confirmed rubella incidence per million persons declined from 172.9 in 2015 to 15.4 in 2016 and 7.1 in 2017. During 2017–2023, the period following the implementation of the catch-up MR vaccination SIA in 2016, the average annual measles incidence per million population in Namibia was 4.9 (range: 0.7–10.5) and decreased 97% from the average during 2010–2016, with the total number of reported confirmed cases decreasing from 2698 during 2010–2016 to 113 during 2017–2023 (Table 2). Similarly, the average annual rubella incidence per million population in Namibia was 2.7, with a range from 0.4 to 7.1. The average annual rubella incidence decreased 95.4% from 2010–2016 to 2017–2023 with the total number of confirmed rubella cases decreasing from 885 during 2010–2016 to 52 during 2017–2023; a total of 4 reported rubella cases occurred among WCBA during 2017–2023 (Table 2).

### 3.1. Measles and Rubella Case-Based Surveillance Performance

Measles and rubella case-based surveillance performance in Namibia met the key indicator target for assessing the sensitivity of the system of ≥2 NMFRI annually since 2010 (Table 3). The key indicator target for nationwide case confirmation by laboratory testing of ≥80% of districts reporting cases with a blood specimen annually was met 5 (36%) times during 2010–2023. In recent years since 2020, the surveillance system performance steadily improved; the NMFRI increased from 3.7 to 12.5 and the proportion of districts reporting cases with a blood specimen increased from 18% to 74% during 2021–2023. However, since 2018, the percentage of districts that investigated at least one measles-suspected case with a blood specimen per year was <80%, indicating suboptimal surveillance performance and likely under-reporting of measles and rubella.

### 3.2. Measles Immunity Profile

**Figure 2:**
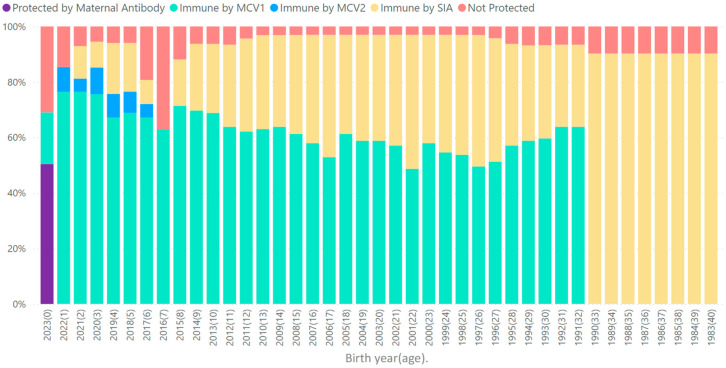
National measles immunity profile as of 31 December 2023, Namibia. Notes: MCV1 = first dose of the measles-containing vaccine; MCV2 = second dose of the measles-containing vaccine; SIAs = supplementary immunization activities. The number of measles-susceptible children under 5 years of age was projected to be 42,954 by 31 December 2023, which was 0.64 times the number of children born in the most recent year.

The estimated number of measles-susceptible children less than 5 years of age was 42,954 as of December 31, 2023, which was 64% of the number of children born in the most recent year, far below the size of one birth cohort, suggesting that population immunity was sufficient to prevent measles outbreaks at that time (Figure 2). Among older children, the highest measles susceptibility remained among those aged 6 years (19%), 7 years (37%), and 8 years (11%), primarily because these birth cohorts were not fully covered by the implemented SIAs. The SIA strategy protected adults born since 1980, reducing measles susceptibility to <10% in each adult birth cohort. For adults born prior to national independence in 1990, MCV1 coverage estimates were not available; therefore, population immunity for those cohorts was based on SIA coverage only and likely underestimated population immunity.

## 4. Discussion

Our analysis showed that Namibia achieved substantial reductions in both measles and rubella incidence following the implementation of elimination strategies, including the introduction of the RCV with the wide age range catch-up SIA in 2016. Since this pivotal SIA, along with the other ongoing elimination strategies, the annual incidence for both measles and rubella has remained <5 per million population, the elimination threshold, and, in fact, at or near zero reported cases. The country has not experienced any large and disruptive measles or rubella outbreaks as it did before 2016. The successful measles campaign in Namibia suggests that sustaining very high (≥ 95%) two-dose MR vaccination rates might not be required to substantially reduce or even interrupt measles and rubella virus transmission in an African context. Further, considering the lower reproductive number of rubella, the measles immunity profile indicated that the EPI, through the use of the MR vaccine, has maintained sufficient levels of population immunity to prevent both measles and rubella outbreaks.

A decline in reported rubella incidence to near zero after the implementation of a wide-age range MR SIA for RCV introduction is consistent with previously described findings in other settings [50,51,52,53]. Prior to the introduction of the RCV in the WHO African Region (AFR), the rubella virus circulated widely throughout the region and primarily infected young children [54]. Estimates from the three largest serosurveys of women of child-bearing age (WCBA) in the AFR prior to the use of the RCV found that rubella susceptibility was 6–16% among WCBA [54]. During 2002–2009 in the AFR, of the 25,631 confirmed rubella cases, 25,097 (98%) had information on age; 3% were infants < 1 year, 28% were 1–4 years, 47% were 5–9 years, 16% were 10–14 years, and 5% were ≥15 years of age, and the mean age of persons with rubella virus infection was 7.3 years [54]. In Namibia during 2002–2009, of the 545 laboratory-confirmed rubella cases, 481 (88.3%) were less than 15 years of age, while 58 (11%) were ≥15 years of age, all in WCBA. Sentinel surveillance for congenital rubella syndrome (CRS) has not been established in Namibia; therefore, the dearth of CRS data makes it difficult to fully estimate the impact of RCV use on disease burden. However, the implementation of high-performing case-based surveillance provided data showing that reported rubella cases decreased to ≤10 annually, and there were only four reported rubella cases among WCBA during 2017–2023.

The analysis and triangulation of multiple key data sources including MR vaccination coverage, MR case-based surveillance, detailed measles outbreak investigations, programmatic risk assessments, and serosurveys provided critical evidence to support the decision made by the country to implement the wide age range MR SIA in 2016 [36,37,38,40,41,43,55,56,57]. Before RCV introduction in Namibia, analysis of surveillance data and outbreak investigations for both measles and polio indicated an epidemiological shift in susceptibility to older age groups and outbreaks following virus importations from cross-border population movement with neighboring countries [36,37,38,43,55,56]. Moreover, the 2013 DHS Survey in Namibia documented wide variations in MCV1 coverage among the provinces, leading to geographically clustered measles susceptibility in the population and corresponding to the areas with increased measles incidence during outbreaks, which were identified as areas with high risk in programmatic risk assessments [58].

Some limitations of the data need to be considered when interpreting the results. First, administrative vaccination coverage for both routine immunization services and SIAs can be biased by inaccurate estimates of population denominators and incorrect reporting of doses delivered, resulting in the need for careful interpretation of the coverage data. Since a PCCS was not implemented in 2016 or 2022, the reported administrative SIA coverage could not be triangulated with survey estimates. Second, under-reporting of measles and rubella through surveillance is well documented; therefore, the surveillance data analyses were representative of reported cases and did not account for undetected and unreported cases, including those cases that may have been seen in the private sector but not included in the nationwide surveillance reporting. Third, comparisons of annual measles and rubella case totals and incidences may be inaccurate if the completeness of case reporting varies from year to year. Finally, surveillance performance around the time of the COVID-19 pandemic indicated that the country was not able to fully document measles and rubella cases as was expected, and so the data likely did not reflect the actual epidemiology of these diseases.

To sustain the reduction in measles and rubella incidence and attain the elimination targets, Namibia needs to achieve and maintain high routine immunization coverage with both doses of the MR vaccine and implement timely and high-quality periodic MR follow-up SIAs, and high-quality elimination-standard MR case-based surveillance need to be implemented [13,27]. The identified measles susceptibility remaining among children born during 2015–2017 heightens the risk for outbreaks and may need to be addressed with targeted vaccination activities to sustain elimination. If an outbreak does occur, these cohorts likely will have a higher age-specific incidence since measles epidemiology reflects susceptibility in the population. A similar scenario from susceptible cohorts that were missed by the SIA strategy was observed in a measles outbreak in Dar es Salaam, Tanzania, during 2006–2007 [59,60]; analysis of the surveillance data during the outbreak investigation revealed cohorts missed by previous SIAs likely contributed to the outbreak occurrence and needed to be covered rapidly during local outbreak response immunizations and a subsequent national follow-up SIA using a wide target age group. Like trends widely documented in other countries, vaccination coverage in Namibia was affected during the COVID-19 pandemic, with a flattening of the MCV1 coverage trend during 2020–2022 and a decline in MCV2 coverage in 2021 [61,62]. Surveillance performance was also noted to have dropped in 2021, with only 18% of districts having reported suspected measles or rubella cases. Sensitive surveillance and targeted efforts to close immunity gaps will be critical to prevent large outbreaks of measles or rubella when virus importations occur. As of early 2024, South Africa has not yet introduced the RCV nationwide in the immunization schedule, and sustained rubella virus transmission is ongoing in South Africa [63]. Angola has very low routine vaccination coverage and has been experiencing large measles and rubella outbreaks, posing ongoing risks of virus transmission across borders into Namibia and other countries. Maintaining high-performing case-based surveillance and capacity for rapid outbreak response will be essential for Namibia to quickly confirm cases and identify and address immunity gaps to stop outbreaks and sustain elimination.

Successful implementation of the 2016 wide age range campaign, along with the other ongoing elimination strategies, likely led to the interruption of endemic measles and rubella viruses in the country, with sporadic cases likely related to importations from neighboring countries that remain endemic for both diseases. However, verification of measles and rubella elimination in Namibia will be needed following the WHO-recommended verification framework [27,64]. The WHO verification framework recommends documentation of required evidence through a national verification committee (NVC) report to the regional verification commission (RVC) to define metrics and key evidence for monitoring progress toward the regional goals for measles and rubella elimination [25,26,27,64]. The interruption of endemic rubella or measles virus transmission is defined as at least 12 months without ongoing local transmission. When interruption of transmission is sustained for 36 months, the RVC weighs the available evidence from the NVC by applying the WHO verification framework [65]. Genotypic documentation of measles and rubella virus strains is critical as part of the process of documenting progress towards elimination and as key evidence for the verification of the elimination of measles and rubella. Given the documented progress toward measles and rubella elimination achieved in Namibia, it is recommended that the NVC compile and submit a report to the RVC for verification.

The IA2030 MRSF includes measles and rubella elimination as a goal and provides guidance to countries for planning and implementing measles and rubella elimination strategies [45]. As of the end of 2022, the elimination of endemic rubella has been verified in 93 countries [28], and measles elimination has been verified in 83 countries [66]; however, none of these countries are in the WHO African Region. Of the 19 countries globally remaining without the RCV, 15 are in the African Region and account for nearly the entire global burden of CRS, highlighting the gross inequities of access to the RVC. Efforts should be made to ensure resources are made available to support universal RCV introduction, including in the remaining 15 countries in the African Region. The implementation of the initial nationwide wide age range MR SIA and periodic follow-up MR SIAs to achieve and maintain population immunity to eliminate rubella and CRS is a golden opportunity for building population immunity and has the additional benefit of reducing any remaining measles susceptibility in older children and young adults if included in the MR SIA target age group to achieve and maintain the elimination of both rubella and measles [36,54,67].

Namibia is currently on track to be one of the first countries in the WHO African Region to be verified for achieving the elimination of both measles and rubella. Until all countries in the region introduce the RCV and make substantial progress toward measles and rubella elimination, the risk for virus importations will remain high. Therefore, it is essential the country maintains high population immunity, as well as high-performing case-based surveillance and robust capacity for rapid outbreak response, to detect and stop outbreaks. Efforts by global partners should be accelerated to ensure expedited universal access and use of the RCV to support measles and rubella elimination. Measles and rubella elimination were both achieved in the Region of the Americas (although re-certification for measles elimination is pending after the re-established endemic virus appears to have been interrupted) and the other regions, including the WHO African Region, will also achieve these goals with continued progress like that achieved in Namibia [50,51,52,53].

## Figures and Tables

**Table 1:** National measles and measles–rubella supplementary immunization activities, 2000–2023, Namibia.

Year	Type of SIAs	Target Age Group	Number of Persons Targeted	Number of Persons Vaccinated	Administrative Coverage	Proportion of Districts with ≥95% Coverage	SIA Coverage by Survey
2000	M Follow-up	9–59 months	285,504	257,590	80%	N/A	N/A
2003	M Follow-up	9–59 months	340,000	318,240	94%	N/A	N/A
2006	M Follow-up	9–59 months	353,270	318,905	97%	N/A	N/A
2009	M Follow-up	9–59 months	246,501	256,006	104%	N/A	N/A
2012	M Follow-up	9 months–15 years	972,163	885,259	91%	100%	97%
2016	MR Catch-up	9 months–39 years	1,859,857	1,908,193	103%	77%	N/A
2022	MR Follow-up	9–59 months	309,916	293,705	95%	53%	N/A

Notes: M = measles, MR = measles–rubella, SIAs = supplementary immunization activities, N/A = not available.

**Table 2:** Laboratory-confirmed measles and rubella cases by sex, setting, and age group, Namibia, 2010–2023.

					Setting *				Age Group											
	Time Period	Cases ^†^	Female		Rural		Urban		<1 y		1–4 y		5–9 y		10–14 y		≥15 y		Women of Reproductive Age (15–49 y)	
	Years	n	n	%	n	%	n	%	n	%	n	%	n	%	n	%	n	%	n	%
Measles	2010–2016	2698	1328	49	1124	65	602	35	74	3	708	26	568	21	342	13	707	26	702	29
	2017–2023	113	49	43	31	27	82	73	0	0	44	39	47	42	8	7	3	3	3	3
Rubella	2010–2016	885	465	53	314	39	482	61	2	2	171	21	343	42	197	24	111	13	110	13
	2017–2023	52	27	52	15	29	37	71	0	0	13	30	18	41	8	18	5	11	4	9

Notes: * Setting classification was reported as either urban or rural; the percentages are of those without a missing value. ^†^ Total number of laboratory-confirmed cases detected through the national case-based surveillance system with specimens tested at the national measles reference laboratory using a standard enzyme-linked immunosorbent assay. The national measles and rubella case-based surveillance uses a case definition of rash, fever, and at least one of cough, coryza, and conjunctivitis, to rash and fever; under-reporting of measles and rubella cases is common and well documented.

**Table 3:** Measles and rubella case-based surveillance performance, 2010–2023, Namibia.

Year	% Districts that Investigated at Least One Measles-Suspected Case with a Blood Specimen per Year (Target: ≥ 80%)	Non-Measles Febrile Rash Illness Rate per 100,000 Population (Target: ≥ 2 per Million Population)
2010	82%	22.6
2011	82%	18.9
2012	73%	16.0
2013	59%	6.8
2014	73%	16.5
2015	75%	45.6
2016	85%	10.9
2017	100%	16.9
2018	91%	11.3
2019	76%	7.3
2020	76%	7.8
2021	18%	3.7
2022	68%	5.5
2023	74%	12.5

## Data Availability

Sources for vaccination coverage and population data presented in this analysis were cited. Data for generating the national population immunity profiles were the WHO-UNICEF Estimates of National Immunization Coverage (available at https://immunizationdata.who.int/, accessed on 16 June 2024) and population data from United Nations Population Division (available at https://population.un.org/wpp/, accessed on 16 June 2024).

## References

[1] Vynnycky E., Adams E., Cutts F., Reef S., Navar A., Simons E., Yoshida L.-M., Brown D.W.J., Jackson C., Strebel P. (2016). Using Seroprevalence and Immunisation Coverage Data to Estimate the Global Burden of Congenital Rubella Syndrome, 1996–2010: A Systematic Review. PLoS ONE.

[2] Patel M., Gacic Dobo M., Strebel P., Dabbagh A., Mulders M., Okwo Bele J.-M., Dumolard L., Rota P., Kretsinger K., Goodson J. (2016). Progress Toward Regional Measles Elimination—Worldwide, 2000–2015. MMWR Morb. Mortal. Wkly. Rep..

[3] United Nations General Assembly (2000). United Nations Millennium Declaration.

[4] World Health Organization (2012). Global Vaccine Action Plan 2011–2020.

[5] Strebel P.M., Cochi S.L., Hoekstra E., Rota P.A., Featherstone D., Bellini W.J., Katz S.L. (2011). A World without Measles. J. Infect. Dis..

[6] Durrheim D.N., Crowcroft N.S. (2017). The price of delaying measles eradication. Lancet Public Health.

[7] Robbins F.C. (1983). Prospects for Worldwide Control of Measles: Discussion I. Rev. Infect. Dis..

[8] Dowdle W., Cochi S. (2011). The principles and feasibility of disease eradication. Vaccine.

[9] Thompson K.M., Badizadegan N. (2017). Modeling the transmission of measles and rubella to support global management policy analyses and eradication investment cases. Risk Anal..

[10] International Task Force for Disease Eradication (1993). Recommendations of the International Task Force for Disease Eradication. MMWR Recomm. Rep. Morb. Mortal. Wkly. Rep. Recomm. Rep..

[11] Strebel P.M., Papania M.J., Gastañaduy P.A., Goodson J.L., Plotkin S., Orenstein W., Offit P., Edwards K.M. (2018). Measles Vaccines. Vaccines.

[12] Shattock A.J., Johnson H.C., Sim S.Y., Carter A., Lambach P., Hutubessy R.C.W., Thompson K.M., Badizadegan K., Lambert B., Ferrari M.J. (2024). Contribution of vaccination to improved survival and health: Modelling 50 years of the Expanded Programme on Immunization. Lancet.

[13] WHO Measles and Rubella Strategic Framework: 2021–2030. https://www.who.int/publications/i/item/measles-and-rubella-strategic-framework-2021-2030.

[14] Goodson J.L., Alexander J.P., Linkins R.W., Orenstein W.A. (2017). Measles and rubella elimination: Learning from polio eradication and moving forward with a diagonal approach. Expert Rev. Vaccines.

[15] World Health Organization (2020). Immunization Agenda 2030 (IA2030).

[16] World Health Organization (2022). Measles Outbreak Guide.

[17] Guerra F.M., Bolotin S., Lim G., Heffernan J., Deeks S.L., Li Y., Crowcroft N.S. (2017). The basic reproduction number (R0) of measles: A systematic review. Lancet Infect. Dis..

[18] Rota P., Moss W., Takeda M., de Swart R., Thompson K., Goodson J. (2016). Measles. Nat. Rev. Dis. Primers.

[19] Nakase T., Brownwright T., Okunromade O., Egwuenu A., Ogunbode O., Lawal B., Akanbi K., Grant G., Bassey O.O., Coughlin M.M. (2024). The impact of sub-national heterogeneities in demography and epidemiology on the introduction of rubella vaccination programs in Nigeria. Vaccine.

[20] World Health Organization (2016). Meeting of the International Task Force for Disease Eradication, November 2015. Wkly. Epidemiol. Rec..

[21] Nguku P.M., Sharif S.K., Mutonga D., Amwayi S., Omolo J., Mohammed O., Farnon E.C., Gould L.H., Lederman E., Rao C. (2010). An investigation of a major outbreak of Rift Valley fever in Kenya: 2006–2007. Am. J. Trop. Med. Hyg..

[22] Strebel P., Grabowsky M., Hoekstra E., Gay A., Cochi S. (2024). Evolution and Contribution of a Global Partnership against Measles and Rubella, 2001–2023. Vaccines.

[23] Perry R., Gacic Dobo M., Dabbagh A., Mulders M., Strebel P., Okwo Bele J.-M., Rota P., Goodson J. (2014). Progress toward regional measles elimination-worldwide, 2000–2013. MMWR Morb. Mortal. Wkly. Rep..

[24] World Health Organization Global Measles and Rubella Strategic Plan 2012–2020. http://www.who.int/immunization/newsroom/Measles_Rubella_StrategicPlan_2012_2020.pdf.

[25] Regional Committee for Africa (2011). Measles Elimination by 2020: A Strategy for the African Region.

[26] Regional Committee for Africa (2021). Framework for the Implementation of the Immunization Agenda 2030 in the WHO African Region: Report of the Secretariat.

[27] Masresha B.G., Hatcher C., Lebo E., Tanifum P., Bwaka A.M., Minta A.A., Antoni S., Grant G.B., Perry R.T., O’Connor P. (2023). Progress Toward Measles Elimination—African Region, 2017–2021. MMWR Morb. Mortal. Wkly. Rep..

[28] Ou A.C., Zimmerman L.A., Alexander J.P., Crowcroft N.S., O’Connor P.M., Knapp J.K. (2024). Progress Toward Rubella and Congenital Rubella Syndrome Elimination—Worldwide, 2012–2022. MMWR Morb. Mortal. Wkly. Rep..

[29] World Bank (2023). World Bank Country Economy Classification by Income. https://datahelpdesk.worldbank.org/knowledgebase/articles/906519-world-bank-country-and-lending-groups.

[30] United Nations Population Division (2022). World Population Prospects. https://population.un.org/wpp/Download/Standard/Population/.

[31] World Health Organization Organisation Mondiale de la Santé (2020). Rubella vaccines: WHO position paper—July 2020—Note de synthèse: Position de l’OMS concernant les vaccins antirubéoleux. Wkly. Epidemiol. Rec. Relev. Épidémiol. Hebd..

[32] Namibia Ministry of Health and Social Services, Expanded Programme on Immunization (2017). Namibia Strategic Plan for Expanded Programme on Immunization 2018–2022.

[33] World Health Organization (2023). Namibia: WHO and UNICEF Estimates of Immunization Coverage: 2022 Revision. https://cdn.who.int/media/docs/default-source/country-profiles/immunization/2023-country-profiles/immunization_nam_2023.pdf?sfvrsn=c1e915f0_3&download=true.

[34] Biellik R., Madema S., Taole A., Kutsulukuta A., Allies E., Eggers R., Ngcobo N., Nxumalo M., Shearley A., Mabuzane E. (2002). First 5 years of measles elimination in southern Africa: 1996–2000. Lancet.

[35] McLean A. (1995). After the honeymoon in measles control. Lancet.

[36] Shibeshi M.E., Masresha B.G., Smit S.B., Biellik R.J., Nicholson J.L., Muitherero C., Shivute N., Walker O., Reggis K., Goodson J.L. (2014). Measles resurgence in southern Africa: Challenges to measles elimination. Vaccine.

[37] Ogbuanu I.U., Zeko S., Chu S.Y., Muroua C., Gerber S., De Wee R., Kretsinger K., Wannemuehler K., Gerndt K., Allies M. (2014). Maternal, fetal, and neonatal outcomes associated with measles during pregnancy: Namibia, 2009–2010. Clin. Infect. Dis..

[38] Ogbuanu I.U., Muroua C., Allies M., Chitala K., Gerber S., Shilunga P., Mhata P., Kriss J.L., Caparos L., Smit S.B. (2016). Measles outbreak reveals measles susceptibility among adults in Namibia, 2009–2011. S. Afr. Med. J..

[39] World Health Organisation African Regional Office (2014). Measles and Rubella Case-Based Surveillance Data Analysis.

[40] Jonas A., Cardemil C.V., Beukes A., Anderson R., Rota P.A., Bankamp B., Gary H.E., Sawadogo S., Patel S.V., Zeko S. (2016). Rubella immunity among pregnant women aged 15–44 years, Namibia, 2010. Int. J. Infect. Dis..

[41] Cardemil C.V., Jonas A., Beukes A., Anderson R., Rota P.A., Bankamp B., Gary H.E., Sawadogo S., Patel S.V., Zeko S. (2016). Measles immunity among pregnant women aged 15–44 years in Namibia, 2008 and 2010. Int. J. Infect. Dis..

[42] Cardemil C.V., Jonas A., Gerber S., Weldon W.C., Oberste M.S., Beukes A., Sawadogo S., Patel S.V., Zeko S., Muroua C. (2014). Poliovirus immunity among pregnant females aged 15–44 years, Namibia, 2010. J. Infect. Dis..

[43] Goodson J.L., Masresha B.G., Wannemuehler K., Uzicanin A., Cochi S. (2011). Changing Epidemiology of Measles in Africa. J. Infect. Dis..

[44] Burton A., Monasch R., Lautenbach B., Gacic-Dobo M., Neill M., Karimov R., Wolfson L., Jones G., Birmingham M. (2009). WHO and UNICEF estimates of national infant immunization coverage: Methods and processes. World Health Organ. Bull. World Health Organ..

[45] Simons E., Mort M., Dabbagh A., Strebel P., Wolfson L. (2011). Strategic planning for measles control: Using data to inform op-timal vaccination strategies. J. Infect. Dis..

[46] World Health Organization Organisation Mondiale de la Santé (2017). Measles vaccines: WHO position paper—April 2017—Note de synthèse de l’OMS sur les vaccins contre la rougeole—Avril 2017. Wkly. Epidemiol. Rec. Relev. Épidémiol. Hebd..

[47] World Health Organization (2016). Planning and Implementing High-Quality Supplementary Immunization Activities for Injectable Vaccines Using an Example of Measles and Rubella Vaccines: Field Guide.

[48] Namibia Ministry of Health and Social Services; Expanded Programme on Immunization. *Namibia Measles and Measles-Rubella Campaign Technical Reports Reported to the World Health Organization (WHO)*. Presented at technical meeting; WHO African Regional Office: Congo, Brazzaville, 2012–2021.

[49] WHO Regional Office for Africa Office (2015). African Regional Guidelines for Measles and rubella Surveillance. https://www.afro.who.int/sites/default/files/2017-06/who-african-regional-measles-and-rubella-surveillance-guidelines_updated-draft-version-april-2015_1.pdf.

[50] Luce R., Masresha B.G., Katsande R., Fall A., Shibeshi M.E. (2018). The Impact of Recent Rubella Vaccine Introduction in 5 Countries in The African Region. J. Immunol. Sci..

[51] Su Q., Ma C., Wen N., Fan C., Yang H., Wang H., Yin Z., Feng Z., Hao L., Yang W. (2018). Epidemiological profile and progress toward rubella elimination in China. 10 years after nationwide introduction of rubella vaccine. Vaccine.

[52] Castillo-Solorzano C., Marsigli C., Bravo-Alcantara P., Flannery B., Ruiz Matus C., Tambini G., Gross-Galiano S., Andrus J.K. (2011). Elimination of rubella and congenital rubella syndrome in the Americas. J. Infect. Dis..

[53] Centers for Disease Control and Prevention (2010). Progress toward control of rubella and prevention of congenital rubella syndrome—Worldwide, 2009. MMWR Morb. Mortal. Wkly. Rep..

[54] Goodson J.L., Masresha B., Dosseh A., Byabamazima C., Nshimirimana D., Cochi S., Reef S. (2011). Rubella Epidemiology in Africa in the Prevaccine Era, 2002–2009. J. Infect. Dis..

[55] Kidd S., Goodson J., Aramburu J., Morais A., Gaye A., Wannemuehler K., Buffington J., Gerber S., Wassilak S., Uzicanin A. (2011). Poliomyelitis outbreaks in Angola genetically linked to India: Risk factors and implications for prevention of outbreaks due to wild poliovirus importations. Vaccine.

[56] World Health Organization (2006). Outbreak of polio in adults—Namibia, 2006. MMWR Morb. Mortal. Wkly. Rep..

[57] Kriss J., De Wee R., Lam E., Kaiser R., Shibeshi M., Ndevaetela E.-E., Muroua C., Shapumba N., Masresha B., Goodson J. (2017). Development of the World Health Organization Measles Programmatic Risk Assessment Tool Using Experience from the 2009 Measles Outbreak in Namibia. Risk Anal..

[58] The Nambia Ministry of Health and Social Services (MoHSS), ICF International (2014). Namibia Demographic and Health Survey 2013.

[59] Goodson J.L., Perry R.T., Mach O., Manyanga D., Luman E.T., Kitambi M., Kibona M., Wiesen E., Cairns K.L. (2010). Measles outbreak in Tanzania, 2006–2007. Vaccine.

[60] Goodson J., Wiesen E., Perry R., Mach O., Kitambi M., Kibona M., Luman E., Cairns K.L. (2009). Impact of measles outbreak response vaccination campaign in Dar es Salaam, Tanzania. Vaccine.

[61] Harris R.C., Chen Y., Côte P., Ardillon A., Nievera M.C., Ong-Lim A., Aiyamperumal S., Chong C.P., Kandasamy K.V., Mahenthiran K. (2021). Impact of COVID-19 on routine immunisation in South-East Asia and Western Pacific: Disruptions and solutions. Lancet Reg. Health West Pac..

[62] Gaythorpe K.A., Abbas K., Huber J., Karachaliou A., Thakkar N., Woodruff K., Li X., Echeverria-Londono S., Ferrari M., Jackson M.L. (2021). Impact of COVID-19-related disruptions to measles, meningococcal A, and yellow fever vaccination in 10 countries. Elife.

[63] Hong H., Malfeld S., Smit S., Makhathini L., Fortuin M., Motsamai T., Tselana D., Manamela M.J., Motaze N.V., Ntshoe G. (2022). A retrospective 5-year review of rubella in South Africa prior to the introduction of a rubella-containing vaccine. PLoS ONE.

[64] Masresha B., Luce R., Tanifum P., Lebo E., Dosseh A., Mihigo R. (2020). The African Region early experience with structures for the verification of measles elimination—A review. Pan Afr. Med. J..

[65] WHO (2018). Guidance for evaluating progress towards elimination of measles and rubella. Wkly. Epidemiol. Rec..

[66] Minta A.A., Ferrari M., Antoni S., Portnoy A., Sbarra A., Lambert B., Hatcher C., Hsu C.H., Ho L.L., Steulet C. (2023). Progress Toward Measles Elimination—Worldwide, 2000–2022. MMWR Morb. Mortal. Wkly. Rep..

[67] Andrus J.K., de Quadros C.A., Solórzano C.C., Periago M.R., Henderson D.A. (2011). Measles and rubella eradication in the Americas. Vaccine.

